# Proposed strategy after complete LAMN (low-grade appendiceal mucinous neoplasm) resection by different RENAPE units: need for a new consensus?

**DOI:** 10.1515/pp-2025-0037

**Published:** 2025-12-15

**Authors:** Christian Mouawad, Abdelkader Taibi, Olivia Sgarbura, Dahbia Djelil, Frédéric Marchal, Isabelle Sourrouille, Diane Goere, Olivier Glehen, Gwenael Ferron, Fréderic Dumont, Thomas Courvoisier Clement, Cécile Brigand, Koceila Lamine Amroun, Karine Abboud, Laurent Villeneuve, Marc Pocard, RENAPE Group

**Affiliations:** Department of Digestive, Hepatobiliary and Liver Transplantation Surgery, AP-HP, Hôpital de la Pitié-Salpêtrière, Paris, France; Université Paris Cité, CNRS UMR 8175, Inserm UMR-S 1334, NABI, Paris, France; SSPC (Simplification des Soins des Patients Complexes) Clinical Research Unit, UPJV 7518, Université de Picardie Jules-Verne, Amiens, France; Digestive Surgery Department, Dupuytren Limoges University Hospital, Limoges, France; CNRS, XLIM, UMR 7252, University Limoges, Limoges, France; Institut du Cancer de Montpellier, Service de Chirurgie Digestive Oncologique, Montpellier, France; Department of Surgical Oncology, Institut de Cancérologie de Lorraine, Vandoeuvre-les-Nancy, France; CRAN, UMR 7039 Université de Lorraine, CNRS, Vandoeuvre-les-Nancy, France; Department of Surgical Oncology, Gustave Roussy Cancer Campus, Villejuif, France; Department of Digestive, Carcinological and Endocrine Surgery, Hôpital Saint-Louis, AP-HP, Paris, France; Université Claude Bernard Lyon 1, UR3738-Centre pour l’Innovation en Cancérologie de Lyon (CICLY), Lyon, France; Hospices Civils de Lyon, Hôpital Lyon Sud, Service de Chirurgie Digestive, Lyon, France; Department of Surgical Oncology, IUCT Oncopole, Institut Universitaire du Cancer de Toulouse, Toulouse, France; Department of Surgical Oncology, Institut de Cancérologie de l’Ouest, Saint Herblain, France; Department of Surgical Oncology, CHU Poitiers, Poitiers, France; Department of General and Digestive Surgery, Hôpital de Hautepierre - Hôpitaux Universitaires, Strasbourg, France; Department of General, Digestive and Endocrine Surgery, Christian Cabrol Hospital University, Reims, France; Department of Surgical Oncology, Hopital Nord St Etienne, St Étienne, France

**Keywords:** low-grade appendiceal mucinous neoplasm, LAMN, RENAPE, surveillance, consensus, national survey

## Abstract

**Objectives:**

To evaluate current postoperative management strategies for incidentally discovered, completely resected low-grade appendiceal mucinous neoplasms (LAMNs) within the French RENAPE network and to assess the need for a new national consensus.

**Methods:**

A national survey was conducted among RENAPE expert centers using a structured questionnaire based on standardized postoperative risk scenarios. Survey items addressed surveillance strategies, indications for reoperation, and use of cytoreductive surgery with hyperthermic intraperitoneal chemotherapy (CRS-HIPEC).

**Results:**

Ninety-one percent of centers responded. All reported systematic multidisciplinary discussion, centralized pathology and imaging review, standardized imaging, and tumor marker assessment. Marked heterogeneity was observed. For low-risk patients (R0 resection, no perforation, no extra-appendiceal mucin), 50 % recommended no follow-up and 50 % proposed long-term MRI surveillance. In intermediate-risk cases (limited perforation or adjacent mucin), 80 % favored MRI follow-up, while 20 % recommended diagnostic laparoscopy or prophylactic CRS-HIPEC. In high-risk scenarios with radiologic suspicion of implants, 60% proposed early CRS-HIPEC, whereas others preferred laparoscopic assessment or surveillance.

**Conclusions:**

This survey highlights wide variation in postoperative strategies for LAMNs within RENAPE, especially in intermediate-risk cases. While consensus exists on centralized review and imaging, significant evidence gaps persist regarding prophylactic HIPEC. These findings underscore the need for harmonized, evidence-based protocols and prospective data collection.

## Introduction

Low-grade appendiceal mucinous neoplasms (LAMNs) are rare epithelial tumors characterized by mucin production and a propensity for peritoneal dissemination, most notably in the form of pseudomyxoma peritonei (PMP) when rupture occurs [1],^2^]. The optimal management of LAMNs, particularly following complete (R0) resection without rupture or peritoneal involvement, remains a subject of ongoing debate due to the scarcity of robust prospective data and the variability in clinical practice [3].

The French RENAPE [4] (Réseau National de prise en charge des Tumeurs Rares du Péritoine) network has played a central role in standardizing the management of rare peritoneal malignancies, including LAMNs. In 2022, the RENAPE network published the French Intergroup Clinical Practice Guidelines for diagnosis, treatment, and follow-up (RENAPE, RENAPATH, SNFGE, FFCD, GERCOR, UNICANCER, SFCD, SFED, SFRO, ACHBT, SFR) for appendiceal tumors and pseudomyxoma peritonei [5]. As an intergroup initiative, these recommendations offer a wide range of treatment options and strategic proposals for defined clinical situations, aiming for consensus but often not providing clear-cut, uniform pathways. Consequently, for many clinical scenarios that do not involve pseudomyxoma peritonei, the guidelines recommend that cases be discussed and managed within a RENAPE expert center, based on expert agreement. However, despite a shared framework, discrepancies persist among RENAPE units regarding postresection surveillance, indications for additional surgical interventions, and the role of hyperthermic intraperitoneal chemotherapy (HIPEC) in certain borderline cases [6].

Some centers advocate for systematic second-look surgery or prophylactic HIPEC in selected patients, particularly those with high-risk features such as acellular mucin on the serosa, perforated appendix, or uncertain resection margins [7], [8], [9]. For example, in the French Intergroup Clinical Practice Guidelines [5], the indication for HIPEC is proposed to be discussed “as an adjuvant” in cases of tumor perforation (spontaneous or iatrogenic) after appendiceal resection, or following an R2 resection with or without associated peritoneal lesions (expert agreement). Such a situation may arise after a LAMN resection performed in a tertiary center. Other expert centers around the world, notably in the United States, the United Kingdom, as well as Italy and France, favor a more conservative approach based on imaging and tumor-marker surveillance, given the generally indolent course of LAMNs and the potential morbidity of overtreatment [10], [11], [12], [13].

This heterogeneity underscores the urgent need to reevaluate current strategies, as it demonstrates how variability in practice can significantly impact daily clinical decision making and highlights the importance of establishing a new consensus to harmonize management across RENAPE units. Such a consensus would aim not only to improve patient outcomes but also to facilitate data pooling for future multicenter studies. In this context, our article explores current postresection strategies among RENAPE centers and discusses whether a unified protocol is warranted considering evolving evidence.

## Materials and methods

To assess the current management strategies for LAMNs following incidental and complete resection, we developed and distributed a structured questionnaire to representatives of multiple RENAPE units across France.

The questionnaire, written in French, was designed around standardized and simplified clinical scenarios that clinicians may encounter after an incidental diagnosis of completely resected LAMN. These scenarios were constructed to reflect varying degrees of risk based on intraoperative findings, histopathological features, imaging results, and biological marker assessments. Scenarios explicitly excluded high-grade appendiceal mucinous neoplasms (HAMNs), R1 resections, and cases with tumor cells present in mucin.

The survey asked participants to specify their management approach (CAT – conduite à tenir) and follow-up strategy for each scenario. It also included sections addressing the routine use of specific clinical information sources such as the following:

– Operative findings (e.g., appendix perforation, presence of mucin on or beyond the appendix)
– Pathological features (e.g., LAMN, perforation, mucin at a distance)
– Confirmation by RENAPATH pathologists
– Imaging (preoperative CT scan, pelvic MRI)
– Tumor marker assays (CEA, CA 19-9, CA 125)

Each RENAPE unit was asked to confirm whether these diagnostic elements were routinely collected and considered in their tumor board discussions. Additionally, participants were queried on specific management plans and timelines for surgery or surveillance depending on risk category and radiological findings.

The questionnaire was sent by email, to designated representatives of the RENAPE network. Responses were received from 91 % of contacted centers (10/11), by a single designated respondent for each center, between February and April 2024 ensuring broad representation of national practices. The complete questionnaire (originally developed in French) used to collect responses from RENAPE centers is provided as Supplementary Material (Appendix 1).

## Results

Responses were received from 91 % of the RENAPE units contacted, providing a comprehensive national overview of current postresection strategies for LAMNs (Table 1). All responding centers reported that cases were routinely reevaluated through multidisciplinary tumor boards, incorporating expert pathology review (RENAPATH) and standardized imaging protocols (RENARAD), particularly postappendectomy abdominopelvic MRI. Additionally, most centers (80 %) reported systematic use of three tumor marker assays (CEA, CA 19-9, and CA 125), whereas a minority (20 %) limited analysis to CEA alone.

**Table 1:** Routine diagnostic practices among RENAPE units.

Routine diagnostic practice	% of centers
Multidisciplinary tumor board review	100 % (10/10)
Standardized imaging protocols (RENARAD)	100 % (10/10)
Tumor markers (CEA, CA 19-9, CA 125)	100 % (10/10)

Management approaches (Table 2) varied depending on postoperative risk stratification. For R0 resected LAMNs with no intraoperative or histological signs of perforation, no mucin outside the appendix, normal imaging, and normal tumor markers, half of the centers proposed no further surveillance, while the other half recommended MRI-based follow-up for up to 10 years, with surveillance intervals ranging from annual to biannual.

**Table 2:** Postresection management strategies (RENAPE network survey).

Clinical scenario	Management strategy	% of centers
Low-risk (R0 resection, no perforation, no extra-appendiceal mucin, normal imaging, and markers)	No further surveillance	50 % (5/10)
	MRI-based follow-up (annual to biannual, up to 10 years)	50 % (5/10)
Intermediate-risk (limited perforation; mucin near appendix, normal imaging, and markers)	MRI follow-up (6 months, then annually)	80 % (8/10)
	Diagnostic laparoscopy within 3 months	10 % (1/10)
	Laparoscopic CRS-HIPEC	10 % (1/10)
High-risk (perforation, mucin aspiration, radiological suspicion of implants)	CRS-HIPEC (laparoscopy, robotic, or laparotomy) within 3–6 months	60 % (6/10)
	Exploratory laparoscopy to confirm extent	30 % (3/10)
	Conservative surveillance	10 % (1/10)

CRS-HIPEC, cytoreductive surgery with hyperthermic intraperitoneal chemotherapy; MRI, magnetic resonance imaging.

In cases with limited perforation (e.g., mucin adjacent to the appendix but no visible peritoneal disease, with normal imaging and tumor markers), the majority (80 %) of centers proposed a radiological follow-up protocol based on pelvic MRI at 6 months and then annually. A small proportion diverged from this approach: 10 % advocated for diagnostic laparoscopy within 3 months, while another 10 % proceeded directly to laparoscopic cytoreductive surgery with HIPEC, reflecting more aggressive strategies.

For patients with perforated LAMN, complete mucin aspiration during surgery, and radiological suspicion of peritoneal implants, 60 % of centers recommended cytoreductive surgery and HIPEC, either via laparoscopy, robotic approach, or laparotomy, typically within 3–6 months after initial surgery. Among the remaining centers, 30 % preferred an exploratory laparoscopy to confirm disease extent before determining treatment strategy, and 10 % recommended a conservative surveillance protocol in the absence of overt progression.

Our initial questionnaire had certain limitations related to the need for clinical scenarios that were simple and easily understandable. As agreed during the design of the cases, we did not include a scenario with an incomplete resection (R1), as described by Arnason et al. [14]. In such situations, some pathology reports emphasize that a new cecal resection is not necessary; however, for patients operated in a tertiary center under emergency conditions, a new laparoscopy could be considered to evaluate for the presence of mucin outside the right quadrant. In line with the same principle of clarity, we also chose not to focus on high-grade appendiceal mucinous neoplasms (HAMN). This limits our study with regard to the ongoing debate about whether or not colonic resection should be performed for this specific tumor type [15].

## Discussion

This national survey highlights both areas of convergence and substantial heterogeneity across RENAPE units regarding postoperative management, particularly in intermediate- and high-risk scenarios. While all centers benefit from multidisciplinary tumor boards, standardized imaging protocols, and centralized pathology review, there remains no unified protocol regarding surveillance intensity or the need for second-look surgery or HIPEC in borderline cases. It is also important to emphasize that the TNM classification for LAMN (Table 3) differs significantly from that of other gastrointestinal tumors, particularly in how local tumor extension and metastatic spread are defined. For LAMN, pTis refers to tumors confined to the appendix, and notably, there are no pT1 or pT2 categories. The pT3 stage describes infiltration into the subserosal tissue, similar to other digestive malignancies. However, the pT4 stage uniquely includes cases with tumor perforation and mucinous deposits limited to the right lower quadrant. Metastatic disease, in contrast, is defined by the presence of tumor deposits beyond the right lower quadrant. Importantly, if mucinous deposits are found close to the appendix, this is not considered a true metastatic situation and should not be classified as pseudomyxoma peritonei (PMP).

**Table 3:** 8th edition AJCC staging of LAMN.

Stage	Definition
pTis (LAMN)	LAMN confined to the muscularis propria
pT3	LAMN or acellular mucin present in the subserosa, but does not extend to serosa
pT4a	LAMN epithelium or acellular mucin present on the serosal surface
M1a	Intraperitoneal acellular mucin
M1b	Intraperitoneal mucinous deposits containing epithelium

AJCC, American Joint Committee on Cancer; LAMN, low-grade appendiceal mucinous neoplasm.

In low-risk patients (R0 resection, no perforation, no extra-appendiceal mucin, and normal markers/imaging), half of the centers opted for long-term MRI surveillance while the other half proposed no follow-up. Recent studies support the safety of conservative management in these settings [16]. Lakmal et al. [11] reported no progression to PMP among patients with nonperforated LAMNs during follow-up, suggesting limited benefit from extended surveillance in this subgroup. Similarly, Hannan et al. [17] reported a progression rate of only 6 % in a structured surveillance program, with no PMP observed in Tis-stage disease, similarly to a study by Wong et al. [18], suggesting that detailed risk stratification can safely reduce follow-up intensity in selected patients.

Intermediate-risk cases, particularly those with localized perforation or acellular mucin near the serosa, elicited varied management strategies. While 80 % of centers favored radiological follow-up, others advocated for exploratory laparoscopy or upfront CRS-HIPEC. This reflects persistent uncertainty regarding the prognostic value of acellular mucin [19], or appendiceal wall perforation [13]. Earlier population-based studies emphasized the association between acellular mucin and PMP development [7], but more recent reviews, including those by the PSOGI and EURACAN working groups, stress that mucin without tumor cells may not warrant invasive treatment [6]. Additionally, the French Intergroup Clinical Practice Guidelines on appendiceal tumors and PMP [5] supported surgical revision in the case of incomplete resection, and adjuvant CRC-HIPEC in case of spontaneous or iatrogenic perforation. Furthermore, in the study by Hannan et al. [17] evaluating a CT-based surveillance regimen, survival analysis showed no statistically significant difference in disease progression with regards to T staging, margin positivity or appendiceal perforation. Conservative management by an MRI-based surveillance strategy in the case of high-risk scenarios was also supported in a case series [10] including histological findings of appendiceal perforation, proximal-margin involvement, and mucinous deposits in 53, 6, and 16.3 % of patients, respectively.

In high-risk scenarios (e.g., perforation with radiologic suspicion of implants), most centers proposed CRS-HIPEC, consistent with international standards of care for PMP [20]. Chua et al. [8] demonstrated that optimal outcomes in PMP hinge on early complete cytoreduction, supporting this aggressive stance. Nonetheless, the preference by some centers for initial diagnostic laparoscopy reflects a cautious approach aimed at accurate disease staging before committing to major cytoreductive surgery, as there is no evidence in the literature that HIPEC improves overall survival or symptom-free survival [16].

The variability we observed mirrors the findings of Govaerts et al. [6], who documented similar disparities in European practice and emphasized the need for unified clinical pathways. While RENAPE has helped centralize expertise, these findings reveal an urgent need to harmonize strategies, particularly for intermediate-risk patients.

Very recently, the German Society of General and Visceral Surgery (DGAV) published the German S2k-guideline on diagnostics, treatment, and surveillance of LAMN [16]. Similar to the French Intergroup Clinical Practice Guidelines, this guideline highlights areas where clear consensus remains lacking, notably for the management of LAMN with perforation. The German guideline states: “LAMN does not metastasize via the lymphatic system or bloodstream; therefore, oncological resections such as right hemicolectomy and lymphadenectomy are not justified. HIPEC and local CRS may be considered as individualized treatments for patients with mucin on the appendiceal surface but should not be performed routinely.”

Some may argue that this persistent heterogeneity in proposed strategies reflects our still limited understanding of LAMN and PMP behavior. Traditionally, analyses have focused only on appendiceal tissue, but emerging evidence suggests that tumor progression in LAMN, low-grade, and high-grade PMP is not driven solely by tumor cells. A recent review underlined that the microbiome differs significantly in these diseases, which could partly explain the variations in clinical evolution observed for similar pathological situations [21].

The strengths of our study include a high response rate and comprehensive representation of expert centers. Limitations include reliance on self-reported practices and standardized scenarios, which may not reflect individualized clinical nuances. Also, self-report and scenario simplification may not capture patient-specific nuance. Furthermore, the absence of patient-reported outcomes or cost-effectiveness analyses limits our ability to contextualize these strategies from a broader health system perspective.

Looking forward, we advocate for a RENAPE-led consensus process, perhaps using a Delphi methodology, to formalize stratified postresection guidelines. Establishing a national registry for resected LAMNs could also enable prospective validation of recurrence risk factors, helping refine surveillance protocols and reduce unnecessary interventions.

In conclusion, while the RENAPE network has succeeded in coordinating high-level care for rare peritoneal tumors, the management of completely resected LAMNs remains inconsistent, particularly in cases with borderline features. The findings of this study underscore the need for updated, evidence-based guidelines to ensure consistent and optimal care delivery across centers.

## Supplementary Material

Supplementary Material
