# Supplementary material for: Proposed strategy after complete LAMN (low-grade appendiceal mucinous neoplasm) resection by different RENAPE units: need for a new consensus?
Source: Pleura Peritoneum. 2025 Dec 15;11(1):49–54. doi: 10.1515/pp-2025-0037 (PMC13001816; doi:10.1515/pp-2025-0037)
Supplement: Supplementary file 1 — Supplementary Material [file j_pp-2025-0037_suppl_001.docx]

# Appendix 1. Questionnaire used in the study

## Management of Low-Grade Appendiceal Mucinous Neoplasms (LAMN) Questionnaire

This questionnaire aims to collect information on the management strategies proposed by RENAPE centers for patients with low-grade appendiceal mucinous neoplasm (LAMN) discovered incidentally after appendectomy.

Please indicate your usual management approach ("CAT – conduite à tenir") for each of the following scenarios. Answers should reflect the practice discussed in your RENAPE multidisciplinary tumor board.

## 1. Information commonly available before discussion

You will usually have access to the following data. Please complete or strike out any items that are not relevant in your practice.

Description by the operating surgeon: (strike out if not applicable)
- Perforated appendix / specimen resected in several fragments
- Mucus outside the appendix, on its surface
- Mucus adjacent to the appendix or in the Douglas pouch, aspirated
- Mucus at distance
- Other: ___________________________

Description by the pathologist: (strike out if not applicable)
- Low-grade appendiceal mucinous neoplasm (LAMN)
- High-grade appendiceal mucinous neoplasm (HAMN)
- Appendiceal adenocarcinoma
- Perforation
- Mucus at distance in specimen
- Other: ___________________________

Confirmation by RENAPATH pathologist:
- Yes, always systematic
- No, not systematic

Preoperative CT scan:
- Yes, to assess for pseudomyxoma peritonei
- No, limited interest due to localized peritonitis

Abdominopelvic MRI (performed in a reference center):
- Presence of pseudomyxoma
- Doubtful image suggesting possible implant

Tumor markers:
- Systematic testing (CEA, CA 19-9, CA 125)
- Only CEA tested
- No systematic testing

## 2. Standardized clinical scenarios

Please propose a single management strategy (CAT) for each scenario. Adaptation to patient-specific factors (age, comorbidities, social circumstances) is assumed.

### Scenario 1 – LAMN with no risk factors

- Normal tumor markers
- No perforation intraoperatively or on histology
- Normal MRI

Proposed management (CAT): _______________________________

If follow-up, duration: _______________________________

### Scenario 2 – LAMN with one risk factor but normal imaging (CT/MRI)

Possible risk factors include:
- Abnormal tumor markers
- Mucus described near appendix (right iliac fossa or pelvic)
- Histologic perforation

Proposed management (CAT): _______________________________

If follow-up, duration: _______________________________

### Scenario 3 – LAMN with risk factors and abnormal MRI (suspicious mucinous lesions)

- Abnormal tumor markers
- Mucus near appendix and aspirated
- Histologic perforation

Proposed management (CAT): _______________________________

If laparoscopy: after what interval? _______________________________

If cytoreductive surgery: after what interval? _______________________________

### Scenario 4 – LAMN with high-risk features and abnormal imaging (identified mucin deposits)

Considered as low-grade pseudomyxoma peritonei.

Proposed management (CAT): _______________________________

*Date of original distribution: February 4, 2024*
